# The Science behind vegetable aromas: types, synthesis, and influencing factors of volatile compounds

**DOI:** 10.1080/15592324.2025.2527958

**Published:** 2025-07-12

**Authors:** Chenxi Ji, Chenxu Liu, Rongqing Wang, Meiying Ruan, Zhuping Yao, Hongjian Wan, Zhimiao Li, Yuan Cheng, Qingjing Ye

**Affiliations:** a College of Horticultural Sciences, Zhejiang A&F University, Hangzhou, PR China; b State Key Laboratory for Quality and Safety of Agro-Products, Vegetable Research Institute, Zhejiang Academy of Agricultural Sciences, Hangzhou, China; c Key Laboratory of Vegetable Germplasm Innovation and Quality Breeding in the Province, Hangzhou, Zhejiang, China; d Xianghu Laboratory, Hangzhou, Zhejiang, China

**Keywords:** Vegetable crops, volatile organic compounds (VOCs), aroma characteristics, biosynthesis, influencing factors

## Abstract

Volatile organic compounds (VOCs) are key determinants of the aroma characteristics in vegetable crops. They not only directly affect consumer sensory experiences but also play important roles in plant – environment interactions. With the continuous advancement of analytical technologies, the composition, biosynthetic pathways, and regulatory mechanisms of VOCs in vegetables have become research hotspots in recent years. This review systematically summarizes the major types of VOCs (aldehydes, esters, terpenes, and sulfur-containing compounds) in vegetable crops and the types of aromas they present. It further explores their biosynthetic pathways, such as those with fatty acids, amino acids, and carotenoids as precursors, as well as the synthesis pathways of terpenoids and phenylpropanoids. In addition, this review covers commonly used enrichment and analytical techniques, including HS-SPME, GC-MS, GC×GC-TOF-MS, as well as the various factors that influence VOC composition – such as genotype, developmental stage, environmental conditions, and postharvest handling. This work provides theoretical support and research directions for the breeding and industrial application of vegetable varieties with improved flavor quality. Future studies should further investigate the functions of VOCs, their regulatory networks, and their roles in plant ecological adaptation.

## 1. Introduction

Volatile organic compounds (VOCs) in vegetable crops not only determine their aroma profiles and are critical factors influencing flavor quality,^1^ but also play important roles in plant communication,^2^ defense against pests and diseases,^3^ responses to abiotic stresses^4^ and human nutrition and health.^5^ In recent years, with the continuous advancement of detection and analytical techniques for VOCs, research on the VOCs of vegetable crops has gradually become a hotspot in the fields of food science, horticulture and ecology.

The types of VOCs in vegetable crops are diverse and their flavor characteristics vary. These compounds mainly include alcohols, aldehydes, ketones, esters and terpenes, which typically exist in plants at low concentrations but can exert a strong sensory impact on humans through the olfactory system.^6^ Different classes of VOCs have distinct flavor characteristics. For example, aldehydes generally possess fatty and green sensory flavor notes. Specifically, 2-octenal provides roasted potato and fatty flavors,^7^ while (E,Z)-2,6-nonadienal and (E)-2-nonenal exhibit cucumber and green aromas.^8^ Terpenes contribute floral and fruity notes to vegetable crops, common examples include linalool and β-ionone, which have floral and woody scents,^9^ and limonene, which has citrus and herbal aromas.^10^ The interaction of different classes of VOCs endows vegetable crops with complex aroma profiles.

These VOCs are synthesized during plant metabolism and are influenced by various factors, including genotype, developmental stage, environmental conditions and postharvest treatments.^11^ Currently, the biosynthetic pathways of VOCs in vegetable crops have been more thoroughly investigated. Studies have shown that the fatty acid metabolism pathway, amino acid metabolism pathway, terpene biosynthesis pathway, and phenylpropanoid metabolism pathway are the primary biosynthetic routes and metabolic processes for plant VOCs.^12^ Given that VOCs are usually present in trace amounts within plants, their qualitative and quantitative analysis requires extremely high sensitivity and accuracy. With the widespread application of detection techniques such as gas chromatography-mass spectrometry (GC-MS), gas chromatography-electronic nose (GC-E-nose), and comprehensive two-dimensional gas chromatography-time-of-flight mass spectrometry (GC×GC-TOF-MS), significant progress has been made in the study of the major VOCs and their biosynthetic regulation in vegetable crops.

Based on these advancements, this review aims to summarize the latest progress in the research on VOCs and aroma characteristics in vegetable crops, explore their composition, metabolic regulation, influencing factors, provide theoretical support for breeding, cultivation and postharvest processing of vegetable crops, thereby promoting the sustainable development of related industries.

## 2. Volatile components of vegetable crops

The volatile substances are mainly composed of esters, aldehydes, terpenes, thiols and other volatile substances. The types and contents of these substances are affected by the types, genotypes and developmental stages of vegetable crops. The difference in the types and contents of volatile substances in different vegetables is the main reason for the diversity of vegetable aroma (Table 1).

**Table 1.** Main aroma components and key substances of common vegetable crops.

Species	Main aroma substance categories	Main aroma components
Vegetable Soybean^13^	Alcohols, aldehydes	1-Octen-3-ol, hexanal, (Z) −2-heptenal, nonanal
Tomato^14^	Aldehydes, ketones	(Z) −3-hexenal, hexanal, 1-octen-3-one, 1-penten-3-one and 3-methylbutanal
Carrots^15^	Terpenoids	sabinene, myrcene, β-caryophyllene and α-humulene
Cabbage^16^	Terpenoids, alcohols, esters	3-mercaptohexanol; 2-propenoic acid, 3-phenyl-, ethyl ester
Celery^17^	Alcohols, esters	4,7-dimethoxy-5-(prop-2-enyl) benzo-1,3-dioxolan or apiole, 3-butylphthalide, 3-butyltetrahydrophthalide or sedanolide
Chinese Chive^18^	ethers	Dimethyl trisulfide
Toona Sinensis^19^	Sulfur-containing compounds, terpenoids	2-Mercapto-3,4-dimethyl-2,3-dihydrothiophene
Mushrooms^20^	Alcohols	1-Octen-3-ol

### 2.1. Aldehydes

Aldehydes are a significant component of the VOCs in vegetables, characterized by their diverse olfactory profiles, ranging from subtle herbal and fruity notes to strong pungent and spicy aromas. These compounds contribute unique sensory attributes to vegetables. Selli et al.^21^ found that aldehydes are the most abundant class of VOCs in fresh cherry tomatoes, with (Z)-3-hexenal and (E)-2-hexenal being the most aroma-active compounds, imparting a strong grassy and green leafy scent to tomatoes. In the case of cucumber, (E,Z)-2,6-nonadienal is a key odorant, providing a violet-like aroma as well as cucumber and melon-like scents.^22^ Additionally, (Z)-9-hexadecenal and (E)-2-tetradecenal, which have citrus and soapy notes, play a crucial role in the distinctive aroma of cilantro.^23,24^ These compounds can help eliminate the gamey odors in meat dishes and enhance appetite. Furthermore, benzaldehyde, 2-octenal and hexanal have been identified as key aroma compounds in enoki mushrooms.^25^ These findings are consistent with our sensory evaluation results, further supporting the link between specific VOCs and sensory preferences.

### 2.2. Ester

Volatile esters impart sweet, fruity, or distinctive aromatic odors to vegetable crops, and some esters may also exhibit a certain degree of bitterness. For instance, ethyl acetate and methyl hexanoate contribute fruity and sweet aromas to vegetables.^26^ Methyl salicylate helps to produce a “wintergreen” and “menthol” scent, while 2,2,4-trimethyl-1,3-pentanediol diisobutyrate exhibits a moldy sensory characteristic and is a major aroma component in potatoes.^27^ Notably, volatile esters are one of the most critical aroma components in chili pepper fruits. Esters such as ethyl 4-methylpentanoate, 3-methylbutyl 2-methylbutanoate and hexyl 2-methylbutanoate play important roles in the formation of the fruity aroma in chili pepper fruits.^28,29^

### 2.3. Terpenoids

Terpenoids are a class of complex compounds, including monoterpenes, sesquiterpenes and diterpenes, which are typically characterized by their strong and distinctive aromas. The monoterpene limonene, a key component in edible fungi, imparts citrus, orange, and fresh notes to mushrooms such as shiitake and truffles.^30,31^ Linalool,^32^ a monoterpene with a lily of the valley scent, adds a refreshing sensory profile to various vegetable crops such as coriander.^24^ α-Pinene, a key aroma compound in potatoes, provides the characteristic turpentine-like odor.^33^ Notably, terpenoids are essential aromatic compounds in carrots, accounting for 98% of the total VOCs in carrots.^34^ Specifically, myrcene and myrtenal contribute green and earthy aromas, while β-caryophyllene and α-humulene provide spicy and woody notes to carrots.^15^

### 2.4. Sulfur compounds

Volatile sulfur compounds are characterized by their strong aroma and low odor thresholds, and they are predominantly found in vegetables with distinctive flavors, such as cruciferous vegetables (broccoli and cabbage) and Allium vegetables (chives, onions and garlic). In cruciferous vegetables, the most important sulfur compounds are glucosinolates (GLS) and their hydrolysis products, isothiocyanates.^35^ The aroma of broccoli and cauliflower is primarily derived from isothiocyanates, which possess a strong pungent and aromatic odor.^36^ In contrast, the main sulfur compounds in Allium vegetables include thiosulfinates, trisulfides and diallyl compounds.^37^ For example, dimethyl trisulfide is the most abundant and odor-contributing compound in chives, and it is also widely present in other Allium species such as garlic, onions and leeks,^38,39^ imparting a garlic and onion-like flavor.^18^ In addition to these, a variety of sulfur compounds, including isothiocyanates, nitriles, epithiosulfonates, thiols, sulfides and polysulfides, are also present in edible mushrooms, collectively determining their unique flavor profiles.^40^

### 2.5. Other volatile substances

In addition to the common VOCs such as esters, aldehydes and terpenes, nitrogen-containing compounds and heterocyclic compounds also play indispensable roles in the formation of vegetable flavors. For example, 2-mercapto-3,4-dimethyl-2,3-dihydrothiophene in Toona sinensis imparts a unique garlic-like aroma,^19^ 2-isobutyl-3-methoxypyrazine provides the distinctive aroma in bell peppers,^41^ and 2-acetyl-1-pyrroline, which has a popcorn-like odor, is also found in some vegetables.^42^ Moreover, organic nitrogen compounds in cabbage^43^are important contributors to its flavor profile.

## 3. Enrichment and identification analysis of volatile substances

### 3.1. Enrichment of volatile substances

The enrichment of VOCs is a crucial step in analysis, involving various methods and techniques. These methods separate volatile components from samples through adsorption, trapping, or distillation to facilitate subsequent analysis. Common enrichment techniques currently in use include solid-phase microextraction (SPME), purge-and-trap (P&T), static headspace (HS), simultaneous distillation-extraction (SDE) and supercritical fluid extraction (SFE).^44^ Each of these techniques has its own advantages and disadvantages and is suitable for different types of samples and target compounds. Among them, Supercritical fluid extraction (SFE) stands out for its ability to control the solvation power of the extractant by optimizing extraction temperature and pressure. Additionally, SFE can selectively separate components from samples by adding organic modifiers to fine-tune the extraction process.^45^ This method has found widespread application in the extraction of vegetable compounds and within the pharmaceutical industry. However, despite its effectiveness, SFE requires specialized equipment and expertise, which may limit its accessibility in some laboratories. P&T is another technique used for VOC enrichment, particularly suitable for identification purposes. However, it falls short in quantitative analysis, restricting its application scope. SDE, while effective, demands a large amount of sample and organic solvent. It is also time-consuming and prone to interference, leading to significant variations in results.^46^ Developing more efficient and resource-saving enrichment techniques to reduce the reliance on a large number of samples and solvents while shortening the analysis time is an important direction of current research. Head-space solid-phase microextraction (HS-SPME) has emerged as the most widely used method due to its combination of SPME with HS techniques. HS-SPME is a rapid, simple, and solvent-free method for volatile extraction, making it highly suitable for the enrichment of VOCs in various vegetable crops, including sweet potatoes,^47^ cabbages,^48^ chili peppers,^49^ matsutake mushrooms^50^ and others.^51^ Although HS-SPME performs well in many applications, its capture efficiency may be insufficient for VOCs with high molecular weight or low volatility. At present, there are relatively few studies on improved methods or new technologies that can efficiently capture this type of VOCs, which is a knowledge gap that needs further exploration. When choosing enrichment techniques, a balance needs to be struck between the universality of the method and its specificity for specific compounds. How to develop enrichment techniques that are both widely applicable and can provide high selectivity under specific circumstances is a challenge in current research.

### 3.2. Detection and analysis of volatile substances

The detection and analysis of VOCs are key steps in studying vegetable flavor. With the continuous development of detection technologies, an increasing number of instrumental analytical methods have been applied to the detection and analysis of VOCs, aiming to obtain more accurate qualitative and quantitative data. Common methods for detecting VOCs mainly include GC-MS, gas chromatography-olfactometry (GC-O), GC-E-nose and GC×GC-TOF-MS. GC-MS is currently one of the most commonly used techniques for detecting VOCs in fruits and vegetables.^52^ It combines the high separation efficiency of gas chromatography with the precise identification capability of mass spectrometry, making it particularly suitable for analyzing complex samples.^53^ However, GC-MS has limitations in resolving co-eluting compounds, which can lead to incomplete identification of VOCs in complex mixtures. Compared with one-dimensional gas chromatography (GC), comprehensive two-dimensional gas chromatography (GC×GC) in GC×GC-TOF-MS can increase signal intensity and peak capacity several to tens of times.^54,55^ This emerging detection method has been mainly applied to the study of VOCs in fruit trees^56,57^ and white spirits,^58,59^ with relatively fewer applications in vegetables. This indicates that there is a knowledge gap in the comprehensive analysis of VOCs in vegetables, and further research is needed to fully utilize the advantages of this technology. GC-O is a technique that combines gas chromatographic separation with human olfactory perception, allowing for the direct evaluation of the olfactory characteristics of VOCs.^60^ GC-E-nose integrates the separation capability of gas chromatography with the rapid detection features of an electronic nose, enabling comprehensive analysis of the overall characteristics of VOCs.^61^ These two techniques offer unique advantages in food flavor research, particularly in identifying key aroma compounds and their corresponding olfactory characteristics. However, their applications in vegetable research are relatively limited, indicating a need for further exploration in this area.

In addition to these methods, many studies have employed multiple detection techniques in combination to analyze the VOCs in vegetable crops. For example, in our previous study, both GC-MS and GC×GC-TOF-MS were used to analyze the VOCs in chili peppers. It was found that different detection methods vary in the number and sensitivity of compounds detected. GC×GC-TOF-MS detected a greater number of VOCs and was more sensitive to hydrocarbons, alcohols and esters, while GC-MS was more sensitive to terpenes.^62^ Dash et al.^63^ detected 104 volatile components in *Piper longum* L. using GC×GC-TOF-MS, including 20 compounds reported for the first time, and separated 24 co-eluting components that could not be resolved using one-dimensional GC columns. Shen et al.^64^ analyzed the VOCs in four types of colored roasted sweet potatoes using HS-SPME-GC-O-MS and HS-SPME-GC×GC-TOF-MS. They found that guaiacol, 1-octen-3-ol, maltol, acetic acid, octanal and β-damascenone were positively correlated with roasted, earthy, caramel, acidic, green and floral notes, respectively, and were the key reasons for the significant differences in flavor among different types of roasted sweet potatoes. Song et al.^65^ used E-Nose, GC-MS/O, GC-IMS and GC×GC-TOF-MS to detect VOCs in yellow mushrooms. They identified key aroma compounds affecting mushroom flavor, such as benzaldehyde and 1-octen-3-one, assessed the olfactory richness (fruity-mushroom and sweet notes) at different developmental stages. Although the combined application of multiple technologies has shown significant advantages in VOCs analysis, there is currently a lack of standardized operation procedures and data integration methods. This leads to the difficulty in directly comparing the results among different studies, limiting the wide promotion of the combined application of multiple technologies. It is suggested that the detection technology be further optimized and research be carried out to establish a standardized operation process and data integration method for the joint application of multiple technologies, so that the results of different studies can be directly compared to better understand the formation mechanism of vegetable flavors.

## 4. Biosynthesis pathway of volatile substances

The biosynthesis of VOCs in vegetable crops is a complex biochemical process involving multiple metabolic pathways and enzymatic catalysis. The primary biosynthetic pathways include the fatty acid metabolism pathway, amino acid metabolism pathway, and the biosynthesis of terpenoids (Table 2). These pathways are interconnected within the plant, collectively regulating the synthesis and release of VOCs.

**Table 2.** Main synthetic pathways, precursor substances and synthetic volatile substance categories.

Synthetic pathway	Key precursor substances	Synthetic volatile substance categories
Biosynthesis pathway with fatty acids as precursors	Linoleic acid and linolenic acid	Aldehydes, esters, alcohols
Biosynthesis pathway with amino acids as precursors	Branched-chain amino acids: leucine, isoleucine and valine Sulfur-containing amino acid: methionine	Branched chain amino acids: alcohols, aldehydes, acids, esters Sulfur-containing amino acids: sulfur-containing volatile substances
Biosynthesis pathway of terpenoids	MVA pathway: acetyl-CoA MEP pathway: pyruvate, glyceraldehyde-3-phosphate	MVA pathway: sesquiterpenes MEP pathway: monoterpenes, diterpenes
Biosynthetic pathway of phenylpropanoids	Phenylalanine	Phenylpropanoids
Biosynthesis pathway with carotenoids as precursors	Carotenoid	Aldehydes, ketones, alcohols

### 4.1. Biosynthesis pathway with fatty acids as precursors

**Figure 1.**
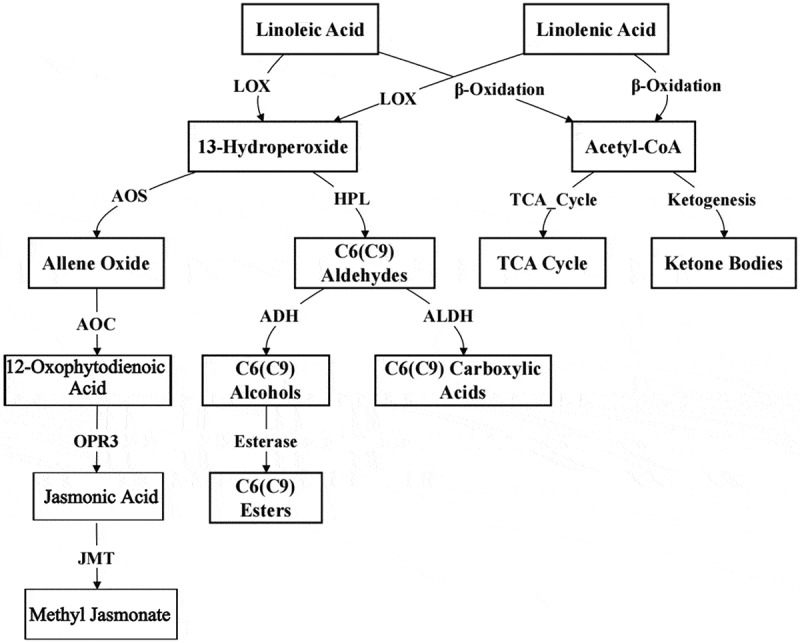
Biosynthesis pathway with fatty acids as precursors.^66^

VOCs derived from fatty acid metabolism primarily originate from the degradation of unsaturated fatty acids, such as linoleic acid and linolenic acid (Figure 1). This pathway is one of the key biosynthetic routes for aldehydes, esters and alcohols in vegetable crops, particularly in Solanaceae family, which includes tomatoes, peppers, and eggplants. Linoleic and linolenic acids are first converted into hydroperoxides by lipoxygenase (LOX).^67^ The hydroperoxides are then processed through a series of reactions. Hydroperoxide lyase (HPL) catalyzes the cleavage of these hydroperoxides, leading to the formation of C9 aldehydes (hexanal, grassy and fatty aroma) and C6 aldehydes (nonanal, sweet and fruity aroma). These aldehydes are important components of plant VOCs. They can be further reduced to the corresponding alcohols by alcohol dehydrogenase (ADH) and subsequently oxidized to carboxylic acids by aldehyde dehydrogenase (ALDH). These compounds can then be esterified by esterases to form esters, thereby increasing the diversity of VOCs. The C6 and C9 aldehydes and alcohols formed in this metabolic pathway are known as green leaf volatiles (GLVs), which impart a fresh, green odor to vegetables and are essential components of vegetable flavor.^68^ In addition to the pathway involving LOX and HPL, linoleic and linolenic acids can also undergo β-oxidation, which converts them into acetyl-CoA. Acetyl-CoA is a versatile molecule that can enter the tricarboxylic acid (TCA) cycle for energy production or participate in the formation of ketone bodies. Moreover, linoleic and linolenic acids are also key precursors for the synthesis of jasmonic acid(JA). Under the action of LOX, linolenic acid is oxidized to 13-hydroperoxide, which is then converted to an epoxide by allene oxide synthase (AOS). Subsequent oxidation by other enzymes generates JA. JA is further methylated by jasmonic acid methyltransferase (JMT) to form methyl jasmonate (MeJA).^69^ These compounds play important roles in plant defense responses, growth and development and the regulation of VOCs.

In summary, the metabolic pathways involving linoleic acid and alpha-linolenic acid are complex and multifunctional. They not only contribute to the aroma and flavor of vegetables by producing VOCs, but also participate in the defense mechanisms and growth regulation of plants. These approaches highlight the complex biochemical processes in the sensory and physiological aspects of plant biology.

### 4.2. Biosynthesis pathways with amino acids as precursors

**Figure 2.**
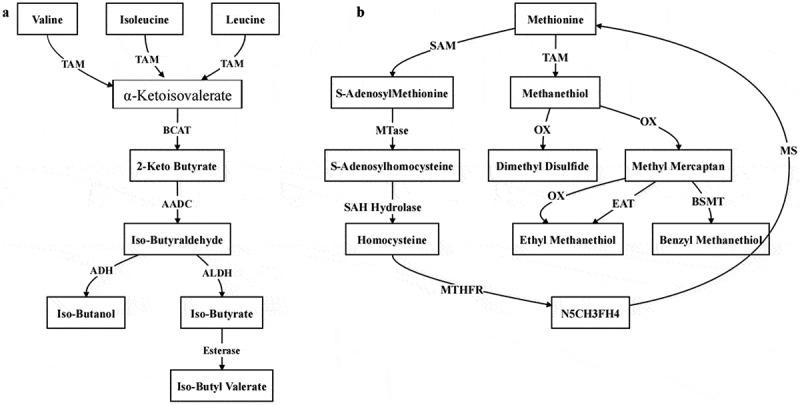
Biosynthesis pathways with amino acids as precursors.^70,71^ (a) branch chain amino acid metabolic pathway;(b) sulfur-containing amino acid metabolic pathway.

The biosynthetic pathways originating from amino acids are primarily divided into branched-chain amino acid (BCAA) derivatives and sulfur-containing amino acid metabolism pathways (Figure 2). In the BCAA pathway, leucine, isoleucine and valine are converted into alcohols through transamination and decarboxylation reactions catalyzed by transaminases and decarboxylases. These alcohols are further oxidized to aldehydes, which are subsequently oxidized to acids, and finally esterified to form volatile esters. VOCs generated via the BCAA pathway can impart fruity, sweet and green notes to vegetables. These compounds are particularly important for the flavor characteristics of certain vegetables. However, excessive levels of these compounds may lead to off-flavors.^72^

In the sulfur-containing amino acid metabolism pathway, methionine is converted to methanethiol by the action of transaminase (TAM). Methanethiol is then oxidized to form sulfur-containing compounds, which are ultimately esterified by esterases to produce volatile sulfur compounds.^72^

These compounds include isothiocyanates, which are renowned for their pungency and unique flavor. These sulfur-containing volatile compounds are particularly important in cruciferous vegetables such as cabbage and mustard, contributing to the unique and characteristic flavors of these plants. Methionine can also be converted to S-adenosylmethionine (SAM) by S-adenosylmethionine synthetase (SAMS). SAM undergoes a series of transfer and hydrolysis reactions to generate homocysteine. Homocysteine can accept a methyl group from N5-methyltetrahydrofolate (N5-CH3-FH4) to regenerate methionine, thus forming the methionine cycle.^73^ This cycle is crucial for the recovery of methionine, ensuring a continuous supply of this amino acid for various metabolic processes.

### 4.3. Biosynthesis pathway of terpenoids

**Figure 3.**
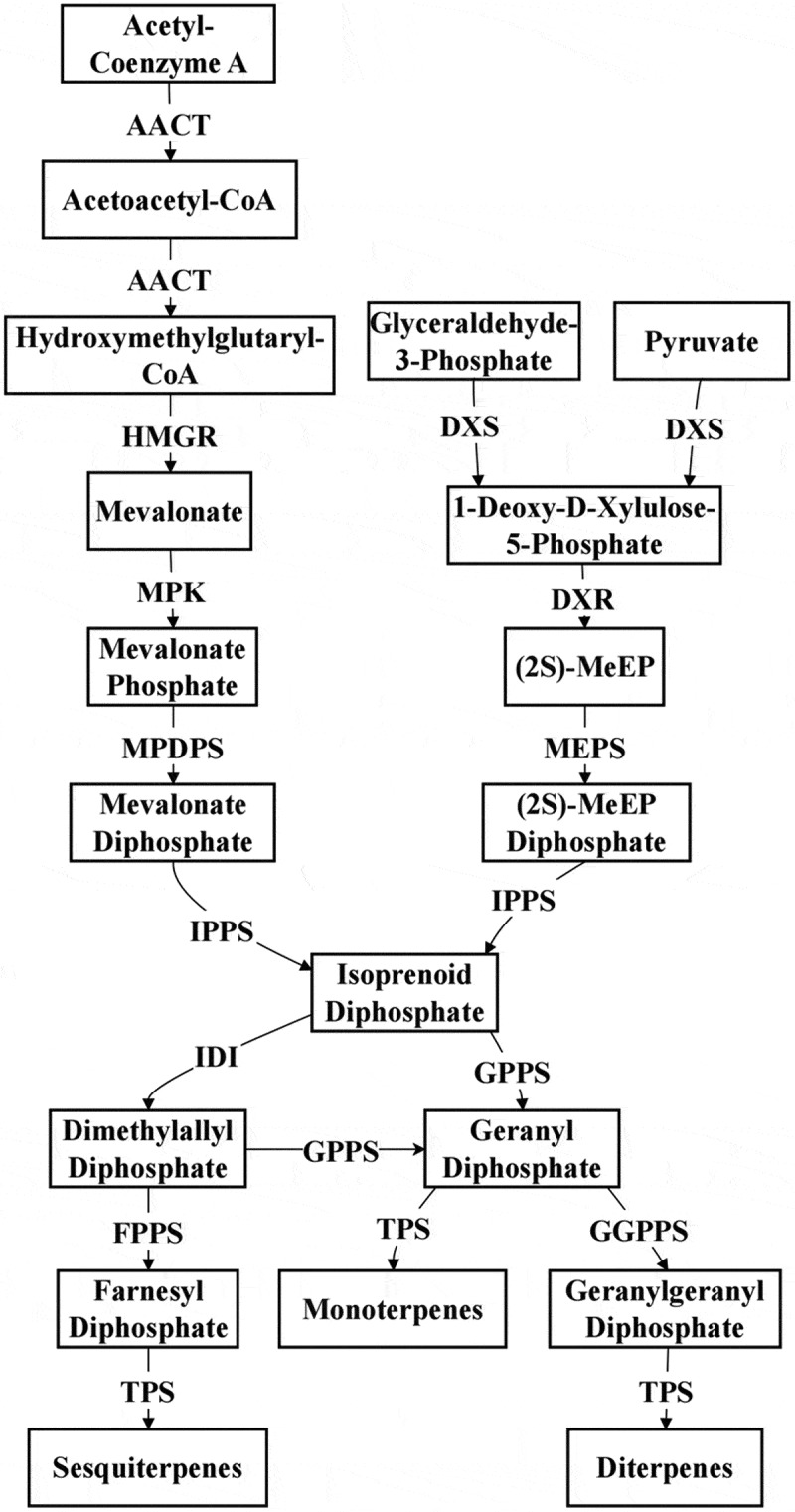
Biosynthesis pathway of terpenoids.^66^

Terpenoids play a critical role in the biosynthesis of VOCs in plants. (Figure 3). The production of terpenoids is primarily governed by two key biosynthetic pathways: the mevalonate (MVA) pathway and the methylerythritol phosphate (MEP) pathway.^74^ The MVA pathway is mainly localized in the cytoplasm, endoplasmic reticulum and peroxisomes,^75^ where it uses acetyl-CoA as the starting substrate. Through a series of enzyme-catalyzed reactions, it generates isoprenoid diphosphate (IPP) and dimethylallyl diphosphate (DMAPP). These intermediates are then utilized in the synthesis of various sesquiterpenoid volatiles.^76^ In contrast, the MEP pathway occurs in the plastids,^77^ with pyruvate and glyceraldehyde-3-phosphate as the initial substrates. After multiple reaction steps, IPP and DMAPP are produced, these are ultimately converted into monoterpenoid and diterpenoid volatiles via terpene synthases (TPS). Notably, IPP and DMAPP serve as universal precursors for terpenoid synthesis, and through various enzymatic reactions, they give rise to a diverse array of terpenoid compounds.^78,79^ This process is fundamental in generating the diverse array of terpenoid VOCs that contribute to the characteristic aromas of plants.

### 4.4. Biosynthesis pathway of phenylpropanoids

**Figure 4.**
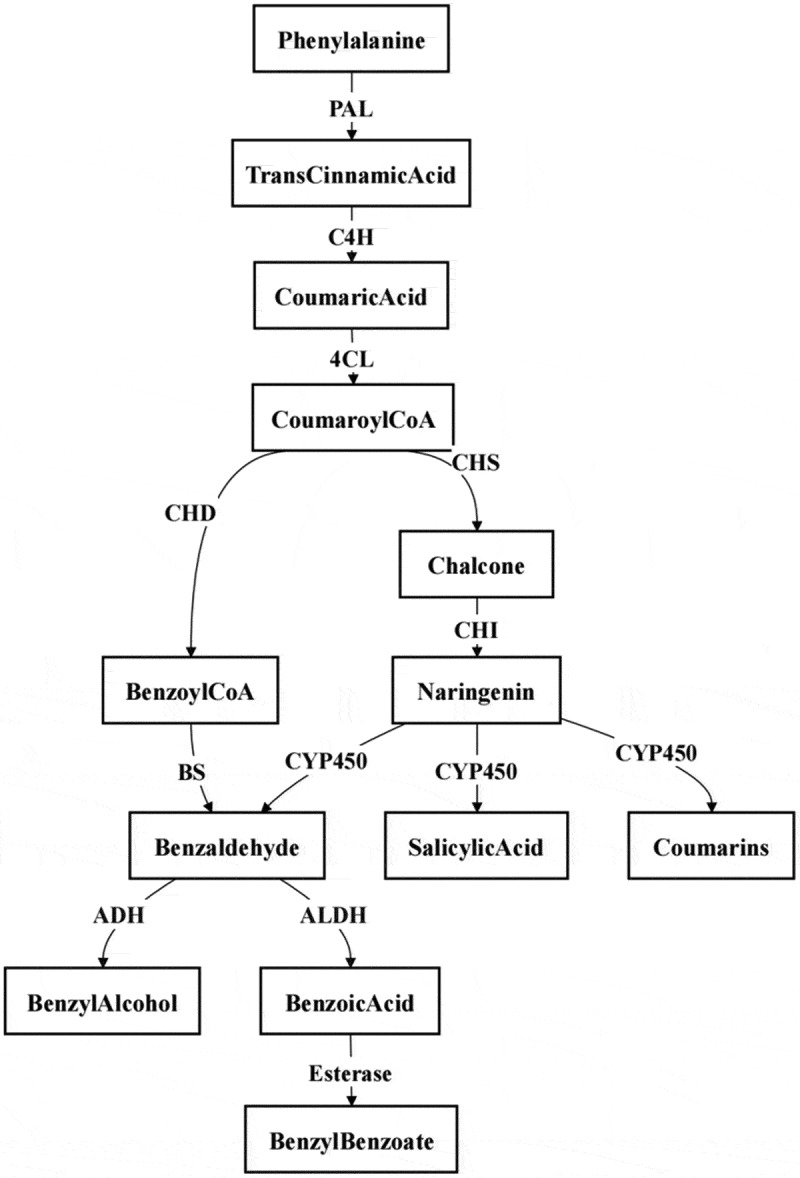
Biosynthetic pathway of phenylpropanoids.^66^

Phenylpropanoids constitute a significant class of secondary metabolites in plants (Figure 4). Phenylalanine serves as the common precursor for volatile phenylpropanoids and aromatic compounds. It is converted to trans-cinnamic acid by the action of phenylalanine ammonia-lyase (PAL). Subsequently, trans-cinnamic acid is hydroxylated to p-coumaric acid by cinnamate 4-hydroxylase (C4H). p-Coumaric acid is then activated to form p-coumaroyl-CoA by the action of 4-coumaroyl-CoA ligase (4CL). Through a series of enzymatic reactions, p-coumaroyl-CoA is converted to naringenin, which is further metabolized to a variety of phenylpropanoid compounds, such as benzaldehyde, salicylic acid and coumarin, by cytochrome P450 monooxygenases (CYP450).^80^ Benzaldehyde can be reduced to benzyl alcohol by alcohol dehydrogenase (ADH) or oxidized to benzoic acid by aldehyde dehydrogenase (ALDH).^81^ Benzoic acid can be esterified to form benzyl benzoate. An alternative route involves the hydrolysis of p-coumaroyl-CoA by cinnamoyl-CoA hydrolase (CDH),^82,83^ resulting in benzoyl-CoA which is directly converted to benzaldehyde by benzaldehyde synthase (BS).^84^ These biochemical pathways not only illustrate the synthesis of phenylpropanoid compounds but also reveal their crucial roles as flavor and aroma precursors in plants. Through the action of these enzymes, plants are able to produce a diverse array of aromatic compounds, which are significant for plant adaptation, defense mechanisms, and attracting pollinating insects.

### 4.5. Biosynthesis pathway with carotenoids as precursors

**Figure 5.**
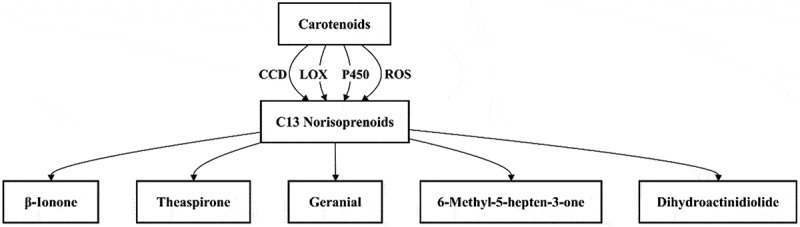
Biosynthesis pathway with carotenoids as precursors.

Carotenoids are not only important pigments in plants but also crucial precursors for the formation of VOCs (Figure 5). When subjected to environmental factors like light and temperature, carotenoids are triggered to undergo oxidative cleavage reactions. This process results in the production of a diverse array of aromatic compounds, including aldehydes, ketones, and alcohols, which significantly contribute to the flavor profiles of vegetables. There are two major degradation pathways: enzymatic oxidation catalyzed by specific carotenoid cleavage dioxygenases (CCDs) or nonspecific enzymes (such as LOX or peroxidases) and non-enzymatic oxidation by reactive oxygen species (ROS).^85^ Both pathways are instrumental in converting carotenoids into VOCs. Under the catalysis of CCD, carotenoids can be converted into C13 derivatives, such as β-ionone and theaflavone, which are VOCs.^86–88^ Additionally, LOX efficiently catalyzes the cleavage of the double bonds in carotenoids, generating aromatic carbonyl compounds. In addition, carotenoids can also be attacked by reactive oxygen species (ROS), forming oxidative intermediates that are further converted into VOCs, adding complexity to the flavor profile of vegetables. The VOCs produced through these pathways are responsible for the impartation of fruity and floral notes to vegetables, enhancing their sensory appeal.

## 5. Influencing factors of volatile substances

**Figure 6.**
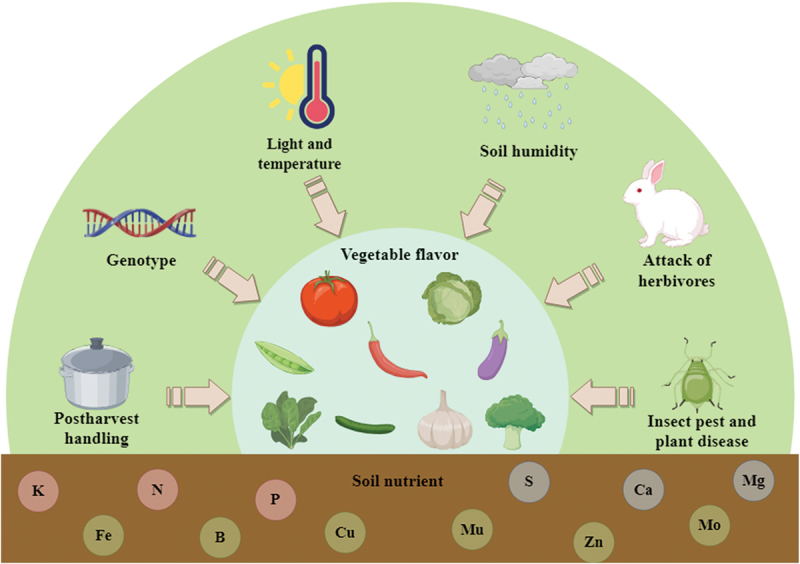
Schematic diagram of influencing factors of vegetable crop flavor.

The composition and concentration of VOCs in vegetable crops are influenced by a variety of factors.^11^ For instance, different genotypes, developmental stages, environmental factors and postharvest treatments can all impact the composition and concentration of plant volatiles (Figure 6).^38,89^

### 5.1. Genotype and transcription factors

The genotype of vegetables significantly influences the types and concentrations of VOCs. Genetic differences lead to distinct volatile profiles. For example, there are significant differences in the types and quantities of VOCs among different varieties and origins of goji berries.^90^ The composition of VOCs varies among carrots of different colors, with white carrots having the highest content of VOCs, followed by orange, purple and yellow carrots.^91^ Sweet potatoes with different flesh colors exhibit significant flavor differences. The aroma of yellow-fleshed sweet potatoes is mainly due to aldehydes such as (E, E)-2,4-heptadienal and nonanal-2,4-decadienal; the sweetness and floral notes in orange-fleshed sweet potatoes may be attributed to carotenoid derivatives such as β-ionone; and the aroma of purple-fleshed sweet potatoes is relatively weak, possibly related to the content and composition of terpenoids.^92^

In recent years, the roles of transcription factors and gene expression levels in the synthesis and regulation of VOCs have garnered significant attention. These elements have been found to be integral to the production and control of VOCs in various vegetables. In tomato fruits, the NAC transcription factor NAC-NOR and the DNA demethylase *SIDML2* play crucial roles in VOC synthesis.^93^ NAC-NOR directly interacts with the promoter region of *SIDML2*, activating its expression. This activation is essential for the regulation of VOC production in tomatoes. Specifically, the binding of NAC-NOR to the *SIDML2* promoter enhances the expression of *SIDML2*, which in turn modulates the synthesis of various VOCs. This regulatory mechanism is vital for the characteristic aroma profile of tomato fruits. Goulet et al.^94^ identified a significant genetic modification in red-fruited tomatoes that affects VOC production. They discovered that the insertion of a retrotransposon in the promoter region of the *SICXE1* gene leads to an increased expression of this gene. The heightened expression of *SICXE1* results in a notable reduction in the ester content of the fruit. This finding underscores the impact of genetic elements on VOC profiles and highlights the potential for genetic modifications to influence fruit flavor. Guo et al.^95^ investigated the genetic basis of VOC production in cucumber plants. They found that specific terpenoid synthase genes, namely SYNTHASE11 (TPS11)/TPS14, TPS01, and TPS15, are responsible for the production of volatile terpenoids in various tissues of cucumber plants, including roots, flowers, and fruits. These terpenoids contribute significantly to the characteristic flavor of cucumbers. The expression of these genes directly influences the production of terpenoid VOCs, thereby enhancing the overall flavor profile of cucumber fruits. The expression pattern of the *CmLOX*s gene in melons is influenced by the orientation properties of the fruit, and it is closely related to the synthesis of esters.^96^ This gene’s expression varies according to the developmental stage and orientation of the melon, affecting the production of ester compounds that are key contributors to the melon’s aroma. The dynamic expression of *CmLOX*s highlights the complexity of VOC regulation in fruits and the importance of gene expression patterns in determining flavor profiles. Liu et al.^97^ conducted comprehensive transcriptomic and metabolomic analyses to explore the regulatory mechanisms of VOC synthesis in tomato fruits. They discovered that transcription factors from the bHLH and AP2/ERF families play pivotal roles in activating the transcription of *SlTNH1*. These transcription factors bind to the promoter sequence of *SlTNH1*, thereby influencing the synthesis of the volatile compound 2-isobutylthiazole. This study emphasizes the importance of transcription factor networks in regulating VOC production and highlights the potential for manipulating these networks to enhance fruit flavor. Another critical regulatory element in VOC synthesis is the HD-Zip family transcription factor, which regulates the *SlLOX* gene in the fatty acid pathway.^97^ This transcription factor modulates the expression of *SlLOX*, thereby affecting the synthesis of hexanal and cis-2-heptenal. These compounds are essential components of the tomato’s aroma, and their production is directly influenced by the activity of the HD-Zip transcription factor. This regulatory mechanism underscores the interconnectedness of metabolic pathways and gene expression in VOCs production.

The synthesis and regulation of VOCs in vegetables are intricately controlled by a network of transcription factors and gene expression patterns. The examples discussed above illustrate the complexity and diversity of these regulatory mechanisms. Understanding these mechanisms is crucial for developing strategies to enhance the flavor profiles of vegetables. Future research should focus on further elucidating the roles of transcription factors and gene expression in VOCs production, as well as exploring the potential for genetic modifications to improve vegetable flavor quality.

### 5.2. Growth and development stages

The developmental stage of vegetable crops significantly influencing their VOCs. As vegetables mature, the types and concentrations of certain VOCs gradually increase, enhancing aroma characteristics and altering the overall flavor profile, which is crucial for determining vegetable freshness. However, over-maturity can degrade VOCs and produce off-flavors. For example, in enoki mushrooms, odor-active compounds are most abundant at the initial fruiting stage, characterized by fruity notes. The concentrations of C8 volatiles and ketones decrease pre-maturity, followed by a slight rise in mushroom and sweet aromas.^65^ The composition of VOCs in celery also changes significantly with maturity, showing increased monoterpenes and sesquiterpenes but decreased phthalates.^98^ During the maturation of melons, the sensory characteristics of fruity and sweet aromas increase, while cucumber-like and sour notes decrease,^99^ clearly indicating ripeness levels.

### 5.3. Growth environment factors

Environmental factors such as light, temperature, water and soil conditions play significant roles in the synthesis and accumulation of VOCs in vegetable crops. Light intensity and duration can influence photosynthesis and respiration in plants, also impact VOC synthesis. For example, the choice of growth substrate significantly affects VOC formation in shiitake mushrooms, with corn cob-based substrates particularly promoting the production of sulfur and C8 volatiles.^100^ Moderate water stress (65% max field water capacity) boosts tomato aroma and quality.^101^ Boron application to roots enhances tomato fruit growth, quality and flavor.^102^ More green light and less red light reduce key flavor compounds and aroma substances like 2-isobutylthiazole in tomatoes.^103^ Bagging treatment can increase the content of key aroma compounds in cucumbers, such as C6 aldehydes, (E,Z)-2,6-nonadienal and (E)-2-nonenal, thereby improving the flavor quality of cucumbers.^104^

Stress responses during the growth and development of vegetables can significantly affect the composition of volatile aroma compounds. When plants encounter biotic or abiotic stresses, they enhance their stress resistance by increasing the synthesis of volatile aroma compounds, which often serve defensive roles, attract pollinators, or inhibit diseases.Biotic stresses, such as herbivory and pathogen attack, can induce significant changes in the volatile profiles of plants. For example, herbivory by insects like aphids can lead to the release of specific VOCs that attract natural enemies of the herbivores, thereby providing a form of indirect defense for the plant. In tomato plants,^105^ herbivory by Manduca sexta larvae has been shown to alter VOC emissions, with significant changes in the levels of monoterpenes, sesquiterpenes, and alkane hydrocarbons. These VOCs not only deter further herbivory but also attract parasitic wasps that prey on the herbivorous insects, thus enhancing the plant’s defense mechanisms. Abiotic stresses, such as salt stress, drought, and temperature extremes, also have profound effects on VOC production. For instance, salt stress has been shown to significantly affect the levels of VOCs in tomatoes,^106^ with the number of VOCs gradually increasing with salinity. Specific compounds such as hexanal and phenylethanol, which contribute to the characteristic aroma of tomatoes, also experience changes in their concentrations under salt stress conditions.Drought stress can lead to the production of specific VOCs that help plants cope with water deficits. For example, water-stressed tomato plants have been found to emit higher levels of α-pinene and methyl salicylate, which are known to play roles in plant defense and signaling.^107^ These VOCs can also influence the behavior of neighboring plants, priming them for enhanced defense responses.

The composition of VOCs in vegetables is intricately linked to the stress responses they undergo during growth and development. Both biotic and abiotic stresses play crucial roles in shaping the volatile profiles of plants, influencing their defense mechanisms, and affecting their overall quality. Understanding these interactions is essential for developing strategies to enhance the flavor and resilience of vegetables. Future research should focus on elucidating the specific mechanisms through which different stress factors influence VOC production and exploring ways to harness these mechanisms for sustainable agricultural practices.

### 5.4. Postharvest handling

The types and concentrations of VOCs in vegetables are significantly influenced by storage conditions, particularly temperature. For example, Xu et al.^108^ found that storing olive vegetables at 25℃ increased the content of aldehydes over time, while storage at 4℃ for 21 days resulted in higher limonene levels, enhancing the fruity aroma. Storage at 0℃ was more effective in preserving the overall composition of volatile compounds. Similarly, the volatile profile of garlic is affected by storage temperature, with the abundance of sulfur compounds like diallyl trisulfide distinguishing fresh garlic from garlic stored for extended periods.^109^ Maul et al.^110^ observed that tomatoes stored at 5℃, compared to those at 20℃, showed a decrease in mature fruit aroma and significant flavor deterioration over time.

Different processing methods also alter the concentration and types of VOCs in vegetables. Bi et al.^111^ discovered that roasting peas increased the variety of key aroma compounds, including pyrazines and pyrazinones, imparting a nutty flavor. Zhang et al.^112^ found that roasting yellow-fleshed sweet potatoes increased the contents of furans and terpenoids, enhancing the overall flavor profile. Nelson et al.^113^ observed significant changes in the concentrations of acetaldehyde, acetone, methanol, and hexanal after heat treatment of tomatoes, with the emergence of methyl mercaptan, a compound not present in fresh tomatoes. Bi et al.^114^ investigated the effects of steaming, frying, boiling, and roasting on garlic, finding that steamed garlic had the richest flavor composition, including the key aroma compound 2-methyl-3-(methylthio)furan. The typical aroma of boiled potatoes originates from methional and various pyrazines produced through the Maillard reaction and Strecker degradation.^115^ Drying temperature can also alter the aroma characteristics of edible fungi; mushrooms dried at 60℃ exhibited more desirable mushroom and almond-like odors.^116^

## 6. Summary and prospect

VOCs hold significant importance in the formation of fruit aroma and quality evaluation in horticultural crops, particularly in fruit and vegetable species, where they have garnered substantial attention. Currently, research on VOCs is relatively advanced in fruit crops such as strawberries^117,118^ and apples,^119^ while studies in vegetables are comparatively limited. This disparity is primarily due to the greater complexity and diversity of VOC types and concentrations in vegetables, which pose significant challenges for research.

With the continuous advancement of detection and analytical techniques, an increasing number of VOCs have been identified in vegetable crops. The identified VOCs play a crucial role in shaping the sensory preferences of consumers. Certain VOCs, such as esters and alcohols, are known to contribute to pleasant and desirable sensory attributes, such as fruity and floral notes. In contrast, other VOCs, like some terpenes, may impart pungent and spicy flavors that are preferred in specific contexts but may be less desirable in others. Understanding the mechanisms by which these VOCs influence sensory perception can help in the development of vegetables with enhanced flavor profiles that better meet consumer preferences. Research on the distribution, flavor characteristics, influencing factors and regulatory molecular mechanisms of these VOCs in different vegetables is still in its infancy. Current studies mainly focus on the identification of VOC types and concentrations, while understanding of their roles in vegetable growth and development, interactions with environmental factors, biosynthetic pathways and regulatory mechanisms remains very limited. These issues continue to be areas that require further investigation.

Future research should focus on integrating genomic, transcriptomic, and metabolomic data to construct a database and odor atlas of VOCs for major vegetable species. This will be achieved through standardized data collection and the development of reference odor maps. At the same time, in-depth analysis of key genes and transcription factors regulating the VOC biosynthetic network is essential. Functional genomics approaches, such as CRISPR/Cas9 genome editing and virus-induced gene silencing (VIGS), should be employed to identify and validate key biosynthetic enzyme genes (e.g., *LOX*, *TPS*, *CCD*) and upstream transcription factors (e.g., *NAC*, *ERF*, *bHLH*). Clarifying their roles in different tissues and developmental stages will facilitate the breeding of vegetables with improved aroma traits through molecular breeding techniques.

Moreover, the development of novel, rapid, and high-throughput platforms for VOC detection and phenotyping is another key area of future research. Current VOC detection methods, such as GC-MS, are accurate but time-consuming and laboratory-dependent. Future research should focus on combining AI-driven data analysis with portable sensor technologies to create rapid and high-throughput VOC phenotyping tools suitable for breeding fields, thereby accelerating the selection of flavor-enhanced varieties. Through these multifaceted studies, a more comprehensive understanding of the formation mechanisms of VOCs in vegetable crops can be achieved, providing a scientific basis for vegetable quality improvement and sustainable agricultural development.

## Supplementary Material

Supplementary materials.docx

## Data Availability

The original contributions presented in the study are included in the article, further inquiries can be directed to the corresponding authors.
