# Supplementary material for: The Science behind vegetable aromas: types, synthesis, and influencing factors of volatile compounds
Source: Plant Signal Behav. 2025 Jul 12;20(1):2527958. doi: 10.1080/15592324.2025.2527958 (PMC12258172; doi:10.1080/15592324.2025.2527958)
Supplement: Supplementary materials.docx [file KPSB_A_2527958_SM3787.docx]

Table S1 Abbreviation of substances and enzymes related to the synthesis pathway of volatile compounds

| Abbreviations | Substance |
| --- | --- |
| Acetyl-CoA | Acetyl-Coenzyme A |
| AACT | Acetoacetyl-CoA Thiolase |
| HMGS | Hydroxymethylglutaryl-CoA Synthase |
| HMG-CoA | Hydroxymethylglutaryl-CoA |
| HMGR | Hydroxymethylglutaryl-CoA Reductase |
| MVA | Mevalonate |
| MPK | Mevalonate Kinase |
| MVAP | Mevalonate Phosphate |
| MPDPS | Mevalonate Diphosphate Synthase |
| MVAPP | Mevalonate Diphosphate |
| IPPS | Isoprenoid Diphosphate Synthase |
| IPP | Isoprenoid Diphosphate |
| IDI | Isoprenoid Diphosphate Isomerase |
| DMAPP | Dimethylallyl Diphosphate |
| FPPS | Farnesyl Diphosphate Synthase |
| FPP | Farnesyl Diphosphate |
| TPS | Terpene Synthase |
| GA3P | Glyceraldehyde-3-Phosphate |
| DXS | 1-Deoxy-D-Xylulose-5-Phosphate Synthase |
| DXP | 1-Deoxy-D-Xylulose-5-Phosphate |
| DXR | 1-Deoxy-D-Xylulose-5-Phosphate Reductase |
| MEP | (2S)-MeEP |
| MEPS | (2S)-MeEP Diphosphate Synthase |
| MEPP | (2S)-MeEP Diphosphate |
| GPPS | Geranyl Diphosphate Synthase |
| GPP | Geranyl Diphosphate |
| GGPPS | Geranylgeranyl Diphosphate Synthase |
| GGPP | Geranylgeranyl Diphosphate |
| KIV | α-Ketoisovalerate |
| BCAT | Branched-Chain Amino Acid Transaminase |
| AADC | Amino Acid Decarboxylase |
| N5CH3FH4 | N5-Methyltetrahydrofolate |
| TAM | Transaminase |
| SAM | S-Adenosylmethionine Synthase |
| MTase | Methyltransferase |
| SAH Hydrolase | S-Adenosylhomocysteine Hydrolase |
| MTHFR | N5-Methyltetrahydrofolate Reductase |
| MS | Methionine Synthase |
| OX | Oxidase |
| BSMT | Benzyl Methylthiol Transferase |
| EAT | Ethyl Methylthiol Transferase |
| PAL | Phenylalanine ammonia lyase |
| C4H | Cinnamate 4-hydroxylase |
| 4CL | Coumaroyl-CoA ligase |
| CHS | Chalcone Synthase |
| CHI | Chalcone Isomerase |
| CYP450 | Cytochrome P450 Monooxygenase |
| CHD | Cinnamoyl-CoA Hydrolase |
| BS | Benzaldehyde Synthase |
| ADH | Alcohol Dehydrogenase |
| ALDH | Aldehyde Dehydrogenase |
| OPDA | 12-Oxophytodienoic Acid |
| JA | Jasmonic Acid |
| MeJA | Methyl Jasmonate |
| LOX | Lipoxygenase |
| AOS | Allene oxide synthase |
| AOC | Allene Oxide Cyclase |
| OPR3 | OPDA Reductase |
| JMT | Jasmonic Acid Methyltransferase |
| HPL | Hydroperoxide lyase |
| CCD | Carotenoid Cleavage Dioxygenase |
| P450 | Cytochrome P450 Monooxygenase |
| ROS | Reactive Oxygen Species |
